# Supplementary material for: Acceleration toward polarization singularity inspired by relativistic E×B drift
Source: Sci Rep. 2016 Nov 24;6:37754. doi: 10.1038/srep37754 (PMC5121894; doi:10.1038/srep37754)
Supplement: Supplementary Information [file srep37754-s1.pdf]

Acceleration toward polarization singularity inspired by relativistic E×B drift

Sunkyu Yu, Xianji Piao, and Namkyoo Park*

Photonic Systems Laboratory, Dept. of Electrical and Computer Engineering, Seoul National University, Seoul 08826, Korea

Supplemental Materials

Supplementary Note 1. The equation of motion for the state of polarization

Starting from the spin-based Hamiltonian equation $d\boldsymbol{\psi}_\mathbf{e}/dz = H_\mathrm{s}\cdot\boldsymbol{\psi}_\mathbf{e}$, the equation of motion for the state of polarization (SOP) [1] can be derived. The Stokes parameters [2] S_j (j = 0,1,2,3) which quantify the SOP can be decomposed with the inclusion of the Pauli matrices, as $S_j = \boldsymbol{\psi}_\mathbf{e}^\dagger\cdot\sigma_j\cdot\boldsymbol{\psi}_\mathbf{e}$. The derivatives of S_j then become

$$\frac{dS_j}{dz} = \frac{d}{dz}\left(\vec{\psi}_e^{\,\dagger}\cdot\sigma_j\cdot\vec{\psi}_e\right) = \frac{d\vec{\psi}_e^{\,\dagger}}{dz}\cdot\sigma_j\vec{\psi}_e + \vec{\psi}_e^{\,\dagger}\sigma_j\cdot\frac{d\vec{\psi}_e}{dz}. \tag{S1}$$

By applying $d\boldsymbol{\psi}_\mathbf{e}/dz = H_\mathrm{s}\cdot\boldsymbol{\psi}_\mathbf{e}$ and $d\boldsymbol{\psi}_\mathbf{e}^\dagger/dz = \boldsymbol{\psi}_\mathbf{e}^\dagger\cdot H_\mathrm{s}^\dagger$ to Eq. (S1), the 'SOP change' operator can be introduced as $S_{\mathrm{d},j} = H_\mathrm{s}^\dagger\cdot\sigma_j + \sigma_j\cdot H_\mathrm{s}$, from the relation of

$$\frac{dS_j}{dz} = \vec{\psi}_e^{\,\dagger}\cdot\left(H_s^{\,\dagger}\sigma_j + \sigma_j H_s\right)\cdot\vec{\psi}_e = \vec{\psi}_e^{\,\dagger}\cdot\hat{S}_{\mathrm{d},j}\cdot\vec{\psi}_e, \tag{S2}$$

where the expectation value of the operator $S_{\mathrm{d},j}$ directly represents the change of each Stokes parameter dS_j/dz.

The Pauli notation of the Hamiltonian $H_\mathrm{s} = a_0\sigma_0 + a_1\sigma_1 + a_2\sigma_2 + a_3\sigma_3$ derives the intuitive expression of the SOP change operator as

$$\begin{aligned}\hat{S}_{\mathrm{d},0} &= H_s^{\,\dagger}\sigma_0 + \sigma_0 H_s = 2\cdot\sum_{j=0}^{3}\mathrm{Re}\left[a_j\right]\cdot\sigma_j \\ \hat{S}_{\mathrm{d},p} &= H_s^{\,\dagger}\sigma_p + \sigma_p H_s \\ &= 2\cdot\mathrm{Re}\left[a_0\right]\cdot\sigma_p + 2\cdot\mathrm{Re}\left[a_p\right]\cdot\sigma_0 - 2\cdot\left\{\mathrm{Im}\left[a_q\right]\cdot\sigma_r - \mathrm{Im}\left[a_r\right]\cdot\sigma_q\right\}\end{aligned}, \tag{S3}$$

where p = 1,2,3, and (p,q,r) is the cyclic order of (1,2,3). The expectation values of Eq. (S3) define the change of S_j in terms of the Stokes parameters as

$$\begin{aligned}\frac{dS_0}{dz} &= 2\cdot\sum_{j=0}^{3}\mathrm{Re}\left[a_j\right]\cdot S_j = 2\cdot\left(\mathrm{Re}\left[a_0\right]\cdot S_0 + \sum_{p=1}^{3}\mathrm{Re}\left[a_p\right]\cdot S_p\right) \\ \frac{dS_p}{dz} &= 2\cdot\mathrm{Re}\left[a_0\right]\cdot S_p + 2\cdot\mathrm{Re}\left[a_p\right]\cdot S_0 - 2\cdot\left\{\mathrm{Im}\left[a_q\right]\cdot S_r - \mathrm{Im}\left[a_r\right]\cdot S_q\right\}\end{aligned}. \tag{S4}$$

Equation (S4) can be expressed in the form similar to the governing equation of electrodynamics [3] as

$$\frac{dS_0}{dz} = 2\cdot \mathrm{Re}[a_0]\cdot S_0 + \mathbf{S}\cdot\mathbf{E}$$
$$\frac{d\mathbf{S}}{dz} = 2\cdot \mathrm{Re}[a_0]\cdot \mathbf{S} + S_0\cdot\mathbf{E} + \mathbf{S}\times\mathbf{B}, \quad \text{(S5)}$$

where $\mathbf{S} = [S_1,S_2,S_3]^T$ is the Stokes vector, and $\mathbf{E} = 2\cdot Re[a_1,a_2,a_3]^T$ and $\mathbf{B} = 2\cdot Im[a_1,a_2,a_3]^T$ each represents the 'pseudo-' electric and magnetic field which drives the Lorentz-like force to the SOP. The condition of $a_0 = 0$ then transforms Eq. (S5) into the simplified form of

$$\frac{dS_0}{dz} = \mathbf{S}\cdot\mathbf{E}$$
$$\frac{d\mathbf{S}}{dz} = S_0\cdot\mathbf{E} + \mathbf{S}\times\mathbf{B}. \quad \text{(S6)}$$

The expressions of the pseudo-fields **E** and **B** are shown as $\mathbf{E} = 2\cdot Im[\varepsilon_1, \varepsilon_2, \varepsilon_3]^T$ and $\mathbf{B} = -2\cdot Re[\varepsilon_1, \varepsilon_2, \varepsilon_3]^T$ for PT-symmetric potentials and as Eq. (4) of the main manuscript for linearly-dichroic chiral materials. By assigning the normalized Stokes parameter [1] as $\mathbf{S_n} = \mathbf{S}/S_0$, Eq. (S6) can also be expressed as $d\mathbf{S_n}/dz = \mathbf{E} + \mathbf{S_n}\times\mathbf{B} - (\mathbf{S_n}\cdot\mathbf{E})\mathbf{S_n}$ (Eq. (2) in the main manuscript).

Supplementary Note 2. Magnetically-induced transition of EP for the achiral eigenstate

We introduce the previously neglected term of $\varepsilon_3\sigma_3$, which can be obtained by imposing an external static magnetic field upon plasmas [3] along the z-axis. The condition of EP ($|\mathbf{B}| = |\mathbf{E}|$) can be maintained by controlling the relative magnitude of the birefringence ($\varepsilon_2\sigma_2$) and the external magnetic field ($\varepsilon_3\sigma_3$). Counterintuitively [4,5], we can achieve the 'achiral' eigenstate at the EP, which can also be controlled by the strength of the external magnetic field for $\varepsilon_3\sigma_3$ (Fig. S1a-c). This active control of the EP eigenstate lifts the restriction on the phenomena near the EP, which have focused only on the chiral state [5-10].

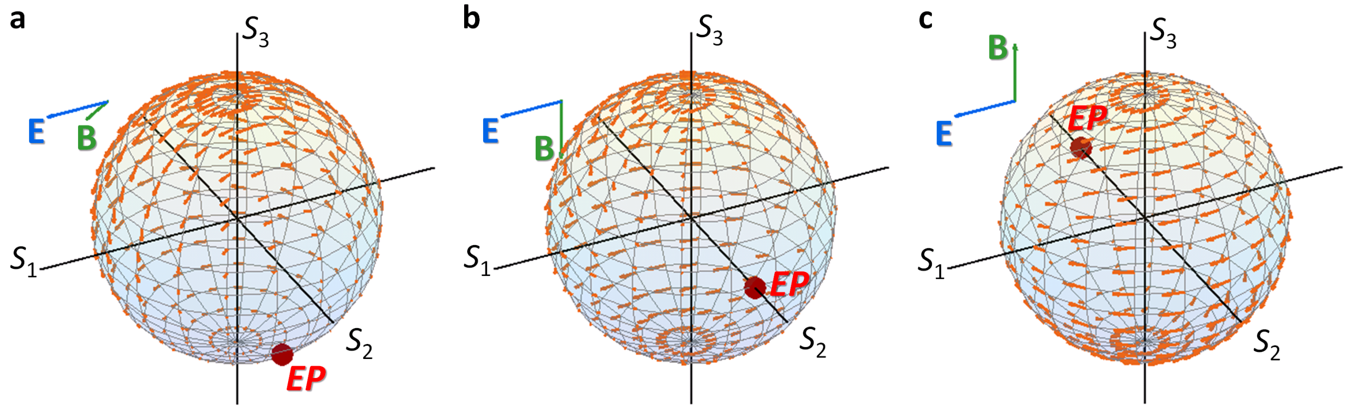

Figure S1. Magnetically-induced transition of EP on the Poincaré sphere. The accelerations at the EP ($|\mathbf{B}| = |\mathbf{E}|$) for different directions of magnetic pseudo-fields: (a) $\mathbf{B} = (\mathbf{e_2} + \mathbf{e_3})/2^{1/2}$, (b) $\mathbf{B} = \mathbf{e_3}$, and (c) $\mathbf{B} = -\mathbf{e_3}$. Red circles denote the EP on the Poincaré sphere, for the SOP of zero Lorentz pseudo-force. The $\mathbf{e_3}$ component of the magnetic pseudo-field originates from the external static magnetic field along the z-axis.

Supplementary Note 3. Derivation of the spin-based Hamiltonian for linearly-dichroic chiral materials

For the z-axis propagating planewave through the chiral material of $\mathbf{\mu} = \mu_0$ and $\varepsilon_x \neq \varepsilon_y$, Maxwell's equations become

$$\begin{aligned}
\frac{\partial E_x}{\partial z} &= -i\omega\cdot(\mu_0 H_y + i\chi E_y) \\
-\frac{\partial E_y}{\partial z} &= -i\omega\cdot(\mu_0 H_x + i\chi E_x) \\
\frac{\partial H_x}{\partial z} &= i\omega\cdot(\varepsilon_y E_y - i\chi H_y) \\
-\frac{\partial H_y}{\partial z} &= i\omega\cdot(\varepsilon_x E_x - i\chi H_x)
\end{aligned}. \quad \text{(S7)}$$

Considering the spatially-varying material along the z-axis, each field has the form of $E_{x,y}(z) = \psi_{\mathrm{e}(x,y)}(z)\cdot exp(-ikz)$ and $H_{x,y}(z) = \psi_{\mathrm{h}(x,y)}(z)\cdot exp(-ikz)$ for the constant k, and Eq. (S7) is then expressed with the field amplitudes $\psi_{\mathrm{e,h}}(z)$ as

$$\begin{aligned}
\psi_{\mathrm{e}x}' - ik\psi_{\mathrm{e}x} &= -i\omega\mu_0\psi_{\mathrm{h}y} + \omega\chi\psi_{\mathrm{e}y} \\
\psi_{\mathrm{e}y}' - ik\psi_{\mathrm{e}y} &= i\omega\mu_0\psi_{\mathrm{h}x} - \omega\chi\psi_{\mathrm{e}x} \\
\psi_{\mathrm{h}x}' - ik\psi_{\mathrm{h}x} &= i\omega\varepsilon_y\psi_{\mathrm{e}y} + \omega\chi\psi_{\mathrm{h}y} \\
\psi_{\mathrm{h}y}' - ik\psi_{\mathrm{h}y} &= -i\omega\varepsilon_x\psi_{\mathrm{e}x} - \omega\chi\psi_{\mathrm{h}x}
\end{aligned}. \quad \text{(S8)}$$

The 4-coupled derivative equations of Eq. (S8) can be transformed to the 2-coupled equations only including the electrical components of $\psi_{\mathrm{e}(x,y)}$. By applying the slowly-varying approximation ($|\psi''| << |k\psi'|$), Eq. (S8) becomes

$$\begin{aligned}
-2ik\cdot\psi_{\mathrm{e}x}' - 2\omega\chi\psi_{\mathrm{e}y}' &= [k^2 - \omega^2\cdot(\mu_0\varepsilon_x - \chi^2)]\cdot\psi_{\mathrm{e}x} + (\omega\chi' - 2ik\omega\chi)\cdot\psi_{\mathrm{e}y} \\
2\omega\chi\cdot\psi_{\mathrm{e}x}' - 2ik\psi_{\mathrm{e}y}' &= (-\omega\chi' + 2ik\omega\chi)\cdot\psi_{\mathrm{e}x} + [k^2 - \omega^2\cdot(\mu_0\varepsilon_y - \chi^2)]\cdot\psi_{\mathrm{e}y}
\end{aligned}. \quad \text{(S9)}$$

We can now separate the derivative terms for x and y polarizations as

$$\begin{aligned}
2\cdot(k^2 - \omega^2\chi^2)\cdot\psi_{\mathrm{e}x}' &= \{ik\cdot[k^2 - \omega^2\cdot(\mu_0\varepsilon_x + \chi^2)] + \omega^2\chi\cdot\chi'\}\cdot\psi_{\mathrm{e}x} \\
&\quad + \{\omega\chi\cdot[k^2 + \omega^2\cdot(\mu_0\varepsilon_y - \chi^2)] + ik\omega\cdot\chi'\}\cdot\psi_{\mathrm{e}y} \\
2\cdot(k^2 - \omega^2\chi^2)\cdot\psi_{\mathrm{e}y}' &= \{-\omega\chi\cdot[k^2 + \omega^2\cdot(\mu_0\varepsilon_x - \chi^2)] - ik\omega\cdot\chi'\}\cdot\psi_{\mathrm{e}x} \\
&\quad + \{ik\cdot[k^2 - \omega^2\cdot(\mu_0\varepsilon_y + \chi^2)] + \omega^2\chi\cdot\chi'\}\cdot\psi_{\mathrm{e}y}
\end{aligned}, \quad \text{(S10)}$$

which leads to the governing Hamiltonian equation for the Cartesian basis as $d[\psi_{\mathrm{e}x},\psi_{\mathrm{e}y}]^{\mathrm{T}}/dz = H_{\mathrm{c}}\cdot[\psi_{\mathrm{e}x},\psi_{\mathrm{e}y}]^{\mathrm{T}}$ where

$$H_{\mathrm{c}} = \frac{1}{2\cdot(k^2 - \omega^2\chi^2)}\times \begin{bmatrix} ik\cdot[k^2 - \omega^2\cdot(\mu_0\varepsilon_x + \chi^2)] + \omega^2\chi\cdot\chi' & \omega\chi\cdot[k^2 + \omega^2\cdot(\mu_0\varepsilon_y - \chi^2)] + ik\omega\cdot\chi' \\ -\omega\chi\cdot[k^2 + \omega^2\cdot(\mu_0\varepsilon_x - \chi^2)] - ik\omega\cdot\chi' & ik\cdot[k^2 - \omega^2\cdot(\mu_0\varepsilon_y + \chi^2)] + \omega^2\chi\cdot\chi' \end{bmatrix}. \quad \text{(S11)}$$

When we apply the spin basis representation using the rotation matrix M as $[\psi_{e+},\psi_{e-}]^T = M\cdot[\psi_{ex},\psi_{ey}]^T$ where

$$M = \frac{1}{\sqrt{2}}\begin{bmatrix} 1 & -i \\ 1 & i \end{bmatrix}, \tag{S12}$$

Eq. (S10) is converted to $d\boldsymbol{\psi}_{\mathbf{e}}/dz = d(M[\psi_{ex},\psi_{ey}]^T)/dz = (MH_cM^{-1})\cdot(M[\psi_{ex},\psi_{ey}]^T) = H_s\cdot\boldsymbol{\psi}_{\mathbf{e}}$ for the $\boldsymbol{\psi}_{\mathbf{e}} = [\psi_{e+},\psi_{e-}]^T$, where the spin-based Hamiltonian H_s becomes $H_s = a_0\sigma_0 + a_1\sigma_1 + a_2\sigma_2 + a_3\sigma_3$ for the Pauli matrices $\sigma_{0,1,2,3}$ and their coefficients,

$$\begin{aligned}
a_0 &= \frac{ik\cdot\{k^2-\omega^2\cdot[\mu_0(\varepsilon_x+\varepsilon_y)/2+\chi^2]\}+\omega^2\chi\cdot\chi'}{2\cdot(k^2-\omega^2\chi^2)} \\
a_1 &= -\frac{ik\omega^2\mu_0\cdot(\varepsilon_x-\varepsilon_y)/2}{2\cdot(k^2-\omega^2\chi^2)} \\
a_2 &= -\frac{\omega^3\chi\mu_0\cdot(\varepsilon_x-\varepsilon_y)/2}{2\cdot(k^2-\omega^2\chi^2)} \\
a_3 &= \frac{i\omega\chi\cdot\{k^2+\omega^2\cdot[\mu_0(\varepsilon_x+\varepsilon_y)/2-\chi^2]\}-k\omega\cdot\chi'}{2\cdot(k^2-\omega^2\chi^2)}
\end{aligned}. \tag{S13}$$

Although Eq. (S13) provides the rigorous explanation for the light-matter interaction in nonmagnetic and electrically anisotropic chiral materials, we consider the simplified case to clarify the physical origin: the preserved sum of ε_x and ε_y along the z-axis ($\varepsilon_x = \varepsilon_o + \Delta\varepsilon(z)$, $\varepsilon_y = \varepsilon_o - \Delta\varepsilon(z)$, and $\chi = \chi_o + \Delta\chi(z)$ where ε_o and χ_o are real). We can then set $k^2 = \omega^2\cdot(\mu_0\varepsilon_o + \chi_o^2)$, and Eq. (S13) becomes

$$\begin{aligned}
a_0 &= \frac{-ik\cdot(2\chi_o\cdot\Delta\chi+\Delta\chi^2)+\chi\cdot\Delta\chi'}{2\cdot(\mu_0\varepsilon_o-2\chi_o\cdot\Delta\chi-\Delta\chi^2)} \\
a_1 &= -\frac{ik\mu_0\cdot\Delta\varepsilon}{2\cdot(\mu_0\varepsilon_o-2\chi_o\cdot\Delta\chi-\Delta\chi^2)} \\
a_2 &= -\frac{\omega\chi\mu_0\cdot\Delta\varepsilon}{2\cdot(\mu_0\varepsilon_o-2\chi_o\cdot\Delta\chi-\Delta\chi^2)} \\
a_3 &= \frac{i\omega\chi\cdot(2\mu_0\varepsilon_o-2\chi_o\cdot\Delta\chi-\Delta\chi^2)-k\cdot\Delta\chi'/\omega}{2\cdot(\mu_0\varepsilon_o-2\chi_o\cdot\Delta\chi-\Delta\chi^2)}
\end{aligned}. \tag{S14}$$

The condition of the constant optical chirality ($\Delta\chi = 0$) derives more explicit form of the coefficients

$$\begin{aligned}
a_0 &= 0 \\
a_1 &= -\frac{ik}{2}\cdot\left(\frac{\Delta\varepsilon}{\varepsilon_o}\right) \\
a_2 &= -\frac{\omega\chi_o}{2}\cdot\left(\frac{\Delta\varepsilon}{\varepsilon_o}\right), \\
a_3 &= i\omega\chi_o
\end{aligned} \tag{S15}$$

and the Hamiltonian H_s becomes $H_s = a_1\sigma_1 + a_2\sigma_2 + a_3\sigma_3$ as shown in the main manuscript.

References for Supplemental Material

[1] H. Kuratsuji and S. Kakigi, Maxwell-Schrödinger equation for polarized light and evolution of the Stokes parameters, Phys. Rev. Lett. **80**, 1888 (1998).
[2] M. C. Teich and B. Saleh, *Fundamentals of photonics* (Wiley Interscience, 2007).
[3] J. D. Jackson, *Classical electrodynamics* (Wiley, 1998), Vol. 3.
[4] W. D. Heiss, The physics of exceptional points, Jour. Phys. A **45**, 444016 (2012).
[5] M. Lawrence, N. Xu, X. Zhang, L. Cong, J. Han, W. Zhang, and S. Zhang, Manifestation of PT Symmetry Breaking in Polarization Space with Terahertz Metasurfaces, Phys. Rev. Lett. **113**, 093901 (2014).
[6] C. Dembowski, B. Dietz, H.-D. Gräf, H. Harney, A. Heine, W. Heiss, and A. Richter, Observation of a chiral state in a microwave cavity, Phys. Rev. Lett. **90**, 034101 (2003).
[7] W. Heiss and H. Harney, The chirality of exceptional points, Eur. Phys. J. D **17**, 149 (2001).
[8] I. Mandal and S. Tewari, Exceptional point description of one-dimensional chiral topological superconductors/superfluids in BDI class, Physica E **79**, 180 (2016).
[9] I. Mandal, Exceptional points for chiral Majorana fermions in arbitrary dimensions, Europhys. Lett. **110**, 67005 (2015).
[10] S. Yu, H. S. Park, X. Piao, B. Min, and N. Park, Low-dimensional optical chirality in complex potentials, Optica **3**, 1025 (2016).
